# Supplementary material for: Structural characterization and inhibition of the interaction between ch-TOG and TACC3
Source: J Cell Biol. 2025 Mar 19;224(6):e202407002. doi: 10.1083/jcb.202407002 (PMC11921806; doi:10.1083/jcb.202407002)
Supplement: Table S1 — shows a summary of HDX data. [file jcb_202407002_tables1.docx]

**Supplementary Table 1: HDX Data Summary Table**

| **Sample** | **TACC3** | **TACC3 + Affimer E4** | **TACC3 + Affimer E7** | **TACC3 + Affimer E8** |
| --- | --- | --- | --- | --- |
| **HDX reaction details** | 8.9 mM Na_2_HPO_4_, 1.5 mM KH_2_PO_4_, 137 mM NaCl, 2.7 mM KCl, pD 7.4 | | | |
| **HDX time course (min)** | 0.5, 5, 30 min | | | |
| **HDX control samples** | Maximally-labeled controls were not performed | | | |
| **Back exchange** | ~ 30% | | | |
| **# of Peptides** | 32 | | | |
| **Sequence coverage** | 63.27% | | | |
| **Average peptide length / Redundancy** | 8.75 / 3.01 | | | |
| **Replicates (biological or technical)** | 3 (technical) | | | |
| **Repeatability (average SD)** | 0.0510 | 0.0454 | 0.0415 | 0.0431 |
| **Significant differences in HDX (Δ HDX > X Da)** | n/a | Hybrid significance test: 99.0% CI: 0.29 Da / p-value < 0.01 | Hybrid significance test: 99.0% CI: 0.29 Da / p-value < 0.01 | Hybrid significance test: 99.0% CI: 0.28 Da / p-value < 0.01 |

SD = standard deviation, CI = confidence interval.
