# Supplementary figures and images for: Structural characterization and inhibition of the interaction between ch-TOG and TACC3
Source: J Cell Biol. 2025 Mar 19;224(6):e202407002. doi: 10.1083/jcb.202407002 (PMC11921806; doi:10.1083/jcb.202407002)

**A**

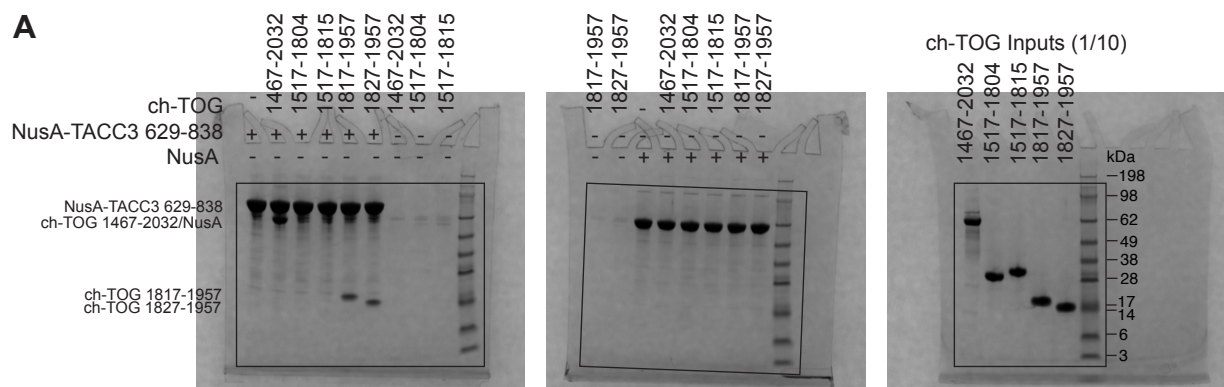

Supplement: SourceData F1 — is the source file for Fig. 1. [file jcb_202407002_sourcedataf1.pdf]

**A**

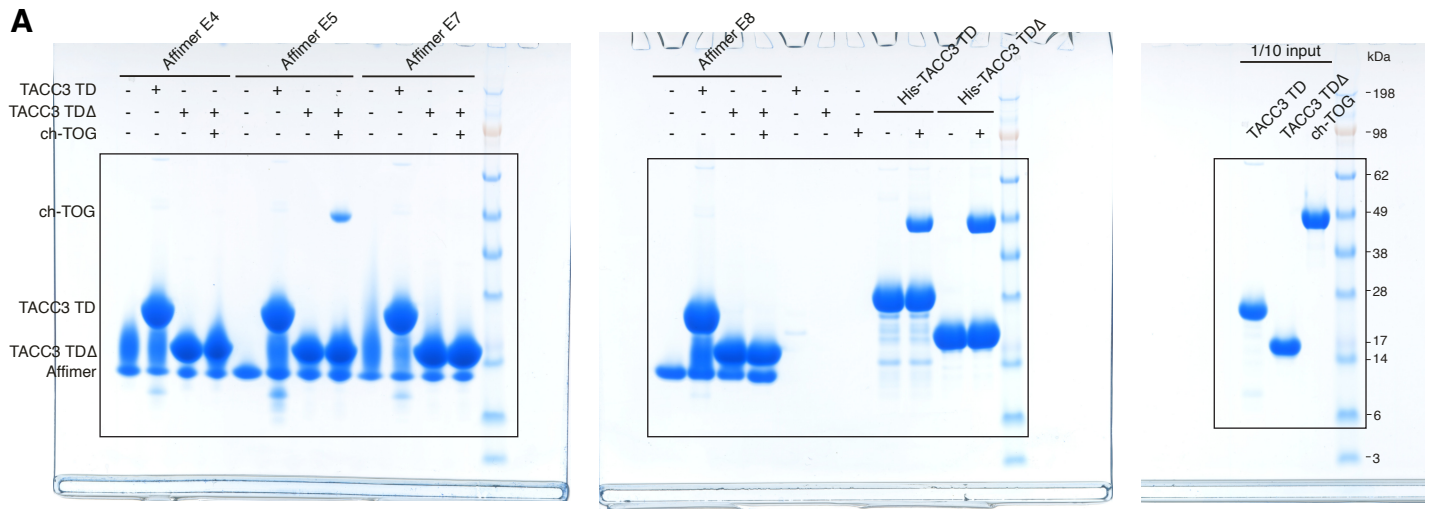

**G**

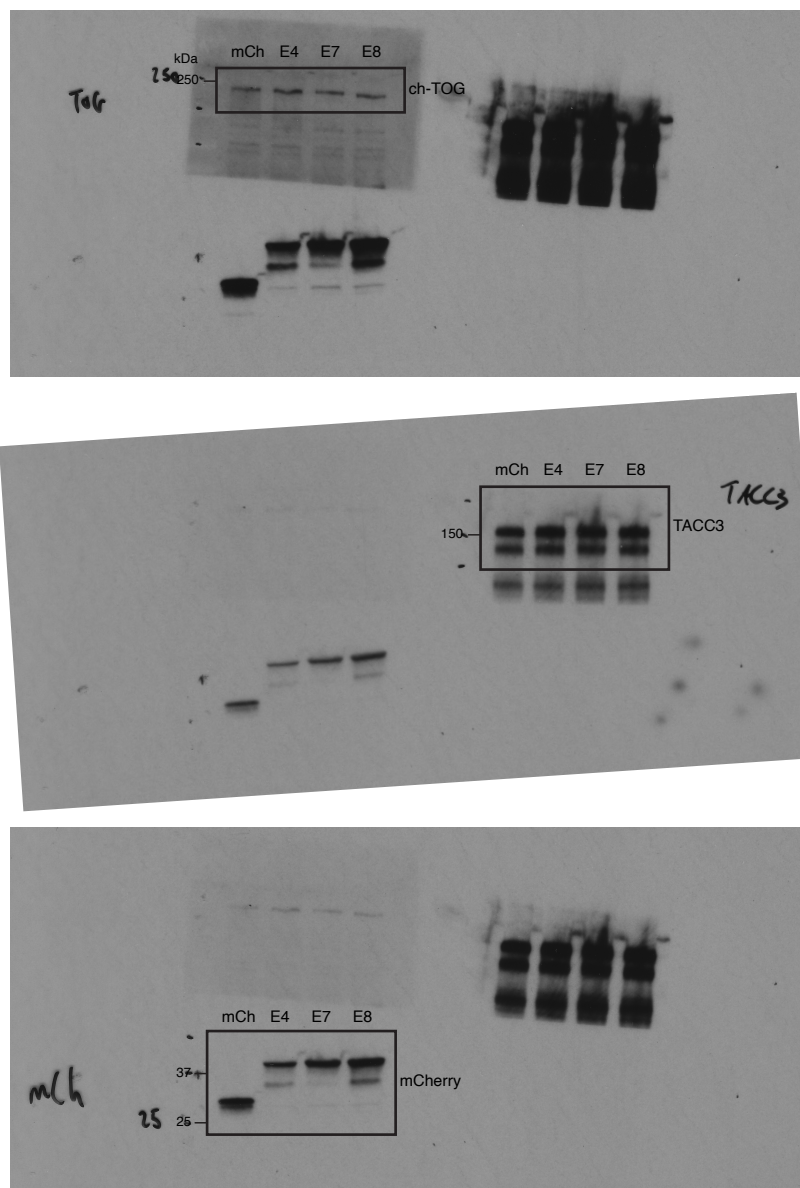

Supplement: SourceData F4 — is the source file for Fig. 4. [file jcb_202407002_sourcedataf4.pdf]

**B**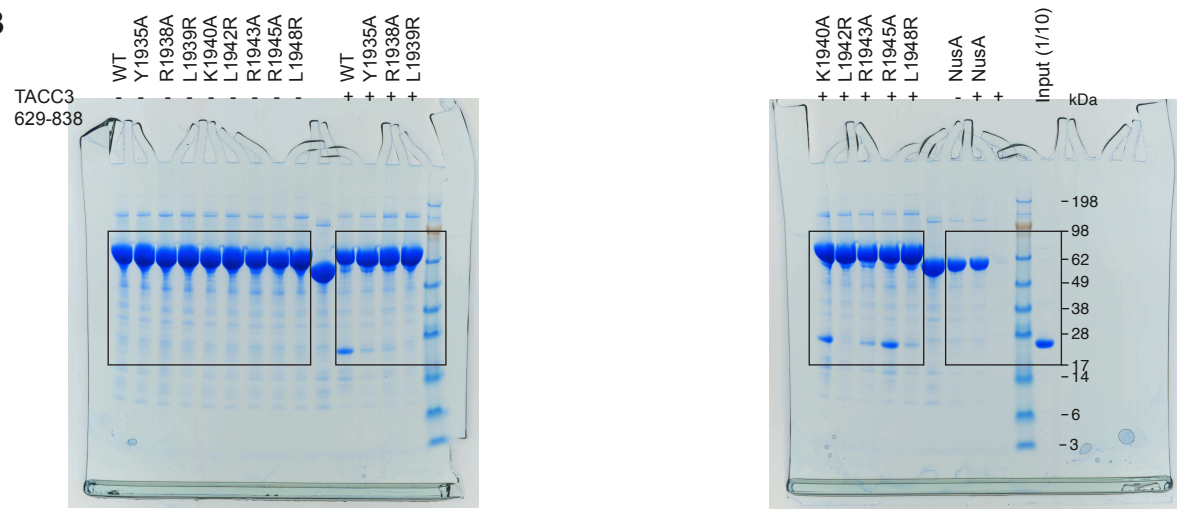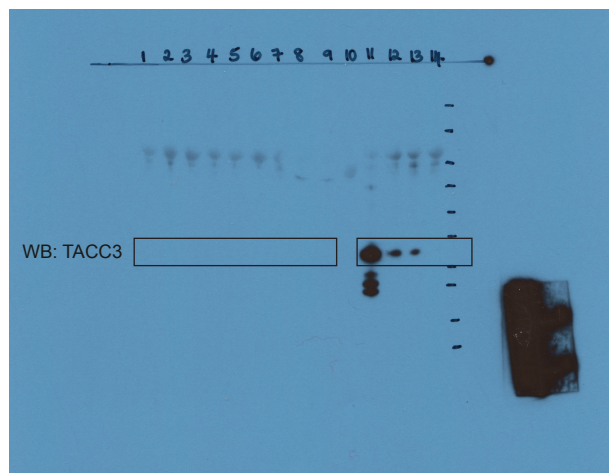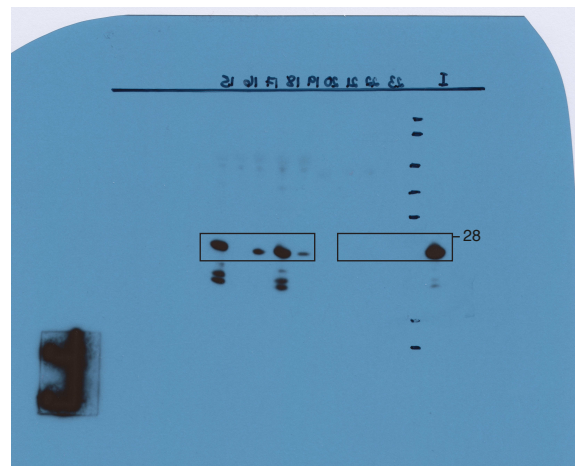

Supplement: SourceData FS3 — is the source file for Fig. S3. [file jcb_202407002_sourcedatafs3.pdf]
